# Diabetes and the Retina: The Role of Medical Treatment

**DOI:** 10.1111/1753-0407.70185

**Published:** 2025-12-22

**Authors:** Zachary Bloomgarden

**Affiliations:** ^1^ Department of Medicine, Division of Endocrinology, Diabetes and Bone Disease Icahn School of Medicine at Mount Sinai New York USA

## Abstract

- About 20% of people with diabetes worldwide have diabetic retinopathy (DR); nearly 25% will have DR in 2045.
- DR and vision impairment are associated with atherosclerotic cardiovascular disease (ASCVD), dementia, and mortality.
- DR tracks with hyperglycemia, and intensive glycemic treatment improves DR, although its ultimate effect in reducing visual impairment is less clear.
- But rapid falls from high to more normal HbA1c are associated with worsening DR.
- BP treatment to systolic levels in the low to mid 140 mmHg range is associated with a reduction in DR, but there is some evidence that systolic levels in the low to mid 120 mmHg range are associated with vision loss.
- A number of classes of medications used for ASCVD improve DR, including inhibitors of the Renin‐Angiotensin‐Aldosterone System (RASi), fenofibrate, and the sodium‐glucose transport (SGLT)‐2 inhibitors.
- It is uncertain whether the glucagon‐like peptide‐1 receptor agonists (GLP‐1RA) are associated with improvement or worsening in DR. This may be particularly true with the more potent agents in this class, perhaps so in the context of rapid and large reductions in HbA1c.

## 1 Introduction

In 2020, diabetic retinopathy (DR) affected 103 million persons worldwide, with projections of 130 million and 161 million in 2030 and in 2045, respectively, of whom, 28% will have vision‐threatening diabetic retinopathy [1]. In the United States, DR causes high prevalence of vision impairment among younger persons, affecting more than twice as many of those age 50–64 with diabetes as those not having diabetes, while among those age 65 or more, the prevalence of vision impairment is approximately 50% greater among those with diabetes [2]. The prevalence of DR in the United States increases from 21% to 54% and 76% among those with diabetes duration < 10, 10–20, and > 20 years, respectively, with proliferative retinopathy (PDR) increasing from approximately 1% to 9% and 32%, and diabetic macular edema (DME) affecting 3%, 13%, and 20% at these durations [3]. DR prevalence increases with increasing levels of blood pressure and of albuminuria [4]. DR is also associated with non‐ocular conditions, with the likelihood of myocardial infarction and of stroke among people with diabetes increasing with worsening degrees of DR [5]. In a meta‐analysis, major adverse cardiovascular event (MACE) and mortality rates doubled among persons with type 2 diabetes (T2D) and quadrupled among those with type 1 diabetes (T1D) having DR, respectively [6]. Individuals with diabetes followed in the Korean National Health Insurance Service database over 15 years showed an association of development of DR with nearly three‐fold multivariate‐adjusted increase in dementia risk [7].

## 2 Effect of Glycemic Control

What medical factors explain the relationship between diabetes and DR? The DETECT‐2 project (Evaluation of Screening and Early Detection Strategies for Type 2 Diabetes and Impaired Glucose Tolerance) was a data‐pooling collaboration evaluating the relationship between glycemic levels and DR across diverse global populations and ethnic groups, finding that the top 20% of the population in levels of glycemia, beginning with fasting glucose ≥ 6.1, 2‐h post‐oral glucose ≥ 11.9, and HbA1c ≥ 6.1%, showed evidence of DR, progressively increasing as glycemic levels increased further [8]. Combined analysis of 35 studies carried out between 1980 and 2008 of 22 896 persons with diabetes showed progressively greater DR prevalence as well as PDR and DME prevalence with increases in HbA1c [3]. Furthermore, treatment of diabetes delays DR development. Prevention of T2D development with lifestyle intervention for persons with pre‐diabetes delayed development of DR in the DaQing study in China [9], and with progressively greater levels of glycemic control there was reduction in development of DR in the Diabetes Control and Complications Trial (DCCT) of persons with T1D [10] and in the United Kingdom Prospective Diabetes Study (UKPDS) [11] and the Action to Control Cardiovascular Risk in Diabetes (ACCORD) trial [12] of persons with T2D. The DCCT, however, showed an important caveat pertaining to glycemic control: comparing those with intensive versus conventional glycemic treatment, with HbA1c goals of 7% versus 9%, respectively, early worsening of DR during the first year of observation occurred nearly twice as commonly, particularly among those with higher HbA1c at the start of the trial and with greater magnitude of HbA1c reduction; although more than half recovered by 18 months in, those with severe pre‐existing retinopathy tended to have more severe worsening of sight‐threatening DR [13].

## 3 Effect of Blood Pressure (BP) Control

BP treatment is another important approach to prevention of DR and its progression. In the UKPDS, those randomized to higher BP (systolic in the 150 s) versus lower BP (systolic in the 140 s) showed no significant difference at 3 years, 37% versus 28% DR progression at 6 years, and 51% versus 34% DR progression at 9 years, with visual deterioration occurring similarly at 3 and 6 years, but reduced by nearly half at 9 years [14]. However, in the ACCORD eye study of persons with T2D, at 4 years there was no effect on progression of DR of systolic BP reduction to 120 versus 140, and there was actually significant worsening of visual acuity in the lower BP group at 8 year follow‐up [15].

## 4 Specific Medications and DR

There is evidence of benefits of RASi, fenofibrate, and SGLT2i, while the question of whether GLP‐1RA are associated with adverse DR outcome has not been fully answered.

## 5 RASi

In a 2‐year study of more than 400 persons with T1D randomized to lisinopril versus placebo, there was no effect on development of new DR, but among those with DR entering the trial progression occurred half as frequently and the development of PDR decreased > 80% [16]. In another study of 285 normotensive patients with T1D and DR, retinopathy progression was one‐third as frequent in those receiving enalapril or losartan compared with those receiving placebo [17]. In a study of 1421 persons with T1D without DR randomized to candesartan versus placebo, there was a 20% reduction in new DR, although a parallel study of 1905 people with T1D and DR failed to show reduction in progression [18].

## 6 Fenofibrate

Fenofibrate reduced DR in two large studies of people with T2D. The Fenofibrate Intervention and Event Lowering in Diabetes (FIELD) study of nearly 10 000 persons randomized to fenofibrate versus placebo showed a 30% reduction in DME and in PDR, with one‐third fewer patients having the composite endpoint of DR progression, DME, or PDR [19]. In the ACCORD study, fenofibrate reduced DR progression by 40%, particularly among those randomized to standard glycemic control that had a 53% reduction in DR progression, while those randomized to intensive glycemic control had a 25% reduction, presumably because of the already lower levels due to better glucose control [12].

## 7 SGLT2i

Although we should recognize the inherent issue of confounding by indication bias to an extent not necessarily addressed by the technique of propensity score matching used in the reviewed studies, there is growing evidence that SGLT2i are associated with improved DR outcomes. Follow‐up of adults with T2D from the Taiwan National Health Insurance Research Database from 2009 to 2020 identified > 200 000 pairs of patients treated with SGLT2i versus dipeptidyl peptidase‐4 inhibitors (DPP‐4i), SGLT2i versus pioglitazone, and SGLT2i versus sulfonylureas, finding SGLT2i to be associated with 43%, 25%, and 38% lower risk of sight‐threatening DR in the respective comparisons [20]. In a study of 7831 propensity score matched pairs treated with empagliflozin versus DPP4i there was no difference in incidence of DR, but a 22% reduction of DR progression among those with existing retinopathy [21].

## 8 Glp‐1RA

We have reviewed aspects of the potential effects of GLP‐1RA on DR previously [22]; considering new studies and the fuller discussion of DR herein, it is reasonable to summarize aspects of the topic.

### 8.1 Pharmacoepidemiological GLP‐1RA Studies Suggesting Adverse DR Effects

Another study from the Taiwan national database compared nearly 100 000 patients with T2D starting either GLP‐1RA or SGLT2i, finding that with prior DR there was a 50% increase in DR progression with GLP‐1RA, or alternatively a 33% decrease in DR progression among those treated with SGLT2i; without prior DR the incidence of new DR was similar [23]. In a study of approximately 2 000 000 people with diabetes receiving insulin using the TriNetX global research network, 176 409 receiving insulin plus SGLT2i and 207 034 receiving insulin plus a GLP‐1RA were compared with insulin‐treated T2D using neither agent, finding SGLT2i use was associated with an 8% increase in DR but a 16% reduction in DME, while GLP‐1RA use was associated with a 31% increase in DR and no reduction in DME [24]. Another TriNetX study using 185 066 persons with T2D treated with a GLP‐1RA propensity‐score matched to non‐GLP‐1RA‐treated patients showed that there was a 7% greater likelihood of developing DR, although with lower likelihood of severe vision loss [25]. Using data from the Danish National Patient Registry of T2D, metformin with basal insulin was associated with a 3.15‐fold greater risk of retinopathy, and metformin with GLP‐1‐RA with a 1.46‐fold increased risk of diabetic retinopathy compared with metformin with DPP‐4i, with metformin with SGLT2i trending to lower rates [26].

### 8.2 Pharmacoepidemiological GLP‐1RA Studies Suggesting DR Benefit

In contrast to the previous studies, a study of 981 patients with diabetes seen at the Cleveland Clinic Cole Eye Institute found that those treated with a GLP‐1RA or SGLT2i showed no difference in DR development or worsening [27]. Another TriNetX database analysis showed significantly lower likelihood of DME with GLP‐1RA, with a similar reduction in patients receiving fibrate treatment [28]. Furthermore, another TriNetX database study compared 185 066 patients with T2D without DR prescribed GLP‐1RA between 2015 and 2022, propensity‐score matched to non‐GLP‐1RA‐treated patients, showing a 7% higher likelihood of developing DR but 1% and 23% lower likelihoods of developing neovascular glaucoma and severe vision loss, and among 32 695 patients with T2D with DR prescribed GLP‐1RA, likelihoods of neovascular glaucoma, panretinal laser phototherapy, vitrectomy, anti‐VEGF tx and severe vision loss were significantly 22%, 21%, 28%, 16%, and 30% lower, respectively [29]. In a retrospective cohort study comparing 810 390 new semaglutide (SGL) users with new users of dulaglutide, empagliflozin, sitagliptin, and glipizide, PDR occurred less frequently with SGL than with the comparators, significantly so compared with glipizide, and treatment‐requiring DR or DME was significantly less frequent with SGL than with dulaglutide, sitagliptin, or glipizide, and did not occur significantly less often with empagliflozin [30].

### 8.3 GLP‐1RA Randomized Controlled Trials (RCT)

Much of the controversy surrounding adverse DR with GLP‐1RA treatment is based on findings from trials of SGL. In studies carried out prior to the cardiovascular outcome trial (CVOT) of SGL, 3.7%–14.5% of participants had DR at baseline, with retinopathy events occurring in 2.1% and 1.5% of those treated with SGL 0.5 and 1.0 mg weekly, as opposed to 2.0% of those receiving placebo, while in the CVOT [29], DR was present at baseline in 29.4% of participants, and DR events occurred in 9.0% and 10.0% of those receiving SGL 0.5 and 1.0 mg weekly, but in 7.6% of those treated with placebo. These DR events were particularly increased in participants with preexisting DR receiving insulin treatment, and this was even more of an issue in those with HbA1c decrease exceeding 1.5%, whether treated with SGL or with placebo, leading a mediation analysis to conclude that the significant 76% greater rate of DR events with SGL was due to the change in HbA1c during the initial 16 weeks of treatment [31]. The conclusion that DR events in RCTs of GLP‐1RA are related to the degree of HbA1c lowering is supported by two other analyses, showing a significant increase in DR only with trials using SGL, but with reduction in MACE also in proportion to the degree of HbA1c lowering, with 77 the number needed to harm from an episode of worsening DR, but with 44 the number needed to treat to avoid one MACE [32, 33]. A further analysis of 23 SGL RCTs involving 22 096 persons with T2D having 730 DR events showed that these particularly occurred at age > 60 and with diabetes duration 10 years or more [34].

Nine RCTs with a total of 10 164 patients have been reported comparing 6700 persons receiving tirzepatide with 3464 receiving placebo; they show a lack of effect on DR [35], but the safety of tirzepatide may be considered less certain inasmuch as participants with PDR, DME, or nonproliferative DR requiring treatment were excluded from the trials.

### 8.4 Summary

DR should be seen both as a complication of diabetes and as a marker of risk of complications including ASCVD and mortality. There is evidence of benefits of glycemic and blood pressure control and evidence that fenofibrate, RASi, and SGLT2i may improve DR. The GLP‐1RA may not have entirely positive effects on DR and perhaps should be initiated gradually in people with underlying DR and marked hyperglycemia, where rapid improvement in HbA1c is to be avoided.

## Funding

The author has nothing to report.

## Conflicts of Interest

The author declares no conflicts of interest.
